# 阿来替尼治疗ALK阳性非小细胞肺癌：1例报告及文献复习

**DOI:** 10.3779/j.issn.1009-3419.2021.101.34

**Published:** 2021-09-20

**Authors:** 海祥 杨, 巍 朱

**Affiliations:** 014040 包头，包头市中心医院肿瘤科 Department of Oncology, Baotou Central Hospital, Baotou 014040, China

**Keywords:** 间变性淋巴瘤激酶, 肺肿瘤, 阿来替尼, 不良反应, Anaplastic lymphoma kinase, Lung neoplasms, Alectinib, Side effects

## Abstract

肺癌是我国乃至全球发病率及死亡率均较高的恶性肿瘤，其中非小细胞肺癌占80%左右，间变性淋巴瘤激酶（anaplastic lymphoma kinase, ALK）基因突变的患者约占5%。ALK抑制剂阿来替尼的疗效优异，药物治疗不良反应的及时发现、及早治疗能极大地提高患者的临床获益。现报道包头市中心医院2020年4月收治的1例ALK阳性非小细胞肺癌的诊断、治疗及药物副反应处理，并文献复习。

## Abstract (en)

Lung cancer is a malignant tumor with high incidence rate and mortality rate in China and even the whole world, of which non-small cell lung cancer accounts for about 80%. Anaplastic lymphoma kinase (ALK) gene mutation accounts for about 5%. Alectinib, ALK-tyrosine kinase inhibitor (ALK-TKI), has great performance in clinical. The early detection and treatment of adverse drug reactions can greatly improve clinical benefits. This paper reports a patient of ALK positive non-small cell lung cancer was admited to Baotou Central Hospital in April 2020. The diagnosis and treatment was retrospectively analyzed, and the literature was reviewed.

现报道包头市中心医院肿瘤科2020年4月收治的1例间变性淋巴瘤激酶（anaplastic lymphoma kinase, ALK）阳性非小细胞肺癌（non-small cell lung cancer, NSCLC）的诊断、治疗及药物副反应处理，并文献复习。

## 1 病例资料

患者女性，60岁，因“气短2月，发现肺占位13天”于2020年4月6日入院。患者于2020年2月初无明显诱因出现气短喘息，患者无发热、盗汗，无胸闷、心悸等不适，患者于2020年3月24日外院胸部电子计算机断层扫描（computed tomography, CT），提示右肺上叶实变影、纵隔淋巴结肿大、胸腔积液，患者为进一步明确右肺上叶实变影就诊于我科，查正电子发射计算机断层显像（positron emission tomography-CT, PET-CT）示（2020年3月31日）：右肺上叶高代谢软组织影，大小约3.0 cm×2.1 cm×2.9 cm，最大标准化摄取值（maximum standardized uptake value, SUVmax）为16.3，考虑恶性，部分层面与领近纵隔胸膜分界不清，右侧锁骨区、纵隔及右肺门多发高代谢肿大淋巴结，考虑转移，右侧胸膜多发局限性增厚伴代谢增高，考虑转移可能。患者无穿刺禁忌，于2020年4月7日行右肺肿物穿刺活检术，术后病理示：肺腺癌，进一步行二代测序（next-generation sequencing, NGS）：*ALK*融合棘皮动物微管相关蛋白4（echinodermmicrotubule-associated protein-like 4, EML4）7%，既往史：2009年诊为乳腺癌行手术治疗、化疗、三苯氧胺内分泌治疗5年，陈旧性肺结核。临床诊断为：右肺上叶恶性肿瘤（cT2N3M1a IVa）（ALK阳性）；淋巴结继发恶性肿瘤；胸膜继发恶性肿瘤；乳腺癌术后、化疗后、内分泌治疗后；陈旧性肺结核。于2020年4月22日开始给予阿来替尼600 mg、每天2次、口服靶向治疗，该患者在用药1周后出现恶心、呕吐，饮食量仅为用药前的1/3，平均每天呕吐1次：2级恶心、1级呕吐，在用药11 d时出现乏力，查血常规：中度贫血：血红蛋白81 g/L，在治疗11 d时给予胃复安5 mg三餐前口服止吐，联合维生素B6、复方谷氨酰胺等药物治疗，患者使用1周后，恶心、呕吐症状好转，进食量逐渐增加到正常，停用胃复安等药物，在治疗过程中未发现椎体外系等相关不良反应，针对患者贫血给予促红细胞生成素（erythropoietin, EPO）10, 000 iu、皮下注射、每周3次，同时加强营养、铁剂、叶酸、维生素B12及抗凝治疗，患者使用2周后，停用EPO治疗过程中未发现血栓形成等不良事件，患者乏力症状明显改善，血红蛋白逐渐增加至110 g/L。患者分别于2020年5月18日及2020年7月20日复查胸部增强CT提示患者右肺上叶前段占位性病变，考虑肺癌（较前明显缩小）；右肺及双侧胸膜、右侧叶间胸膜多发结节影，考虑转移（较前明显缩小）。目前该患者继续口服阿来替尼靶向治疗中。

**图 1:**
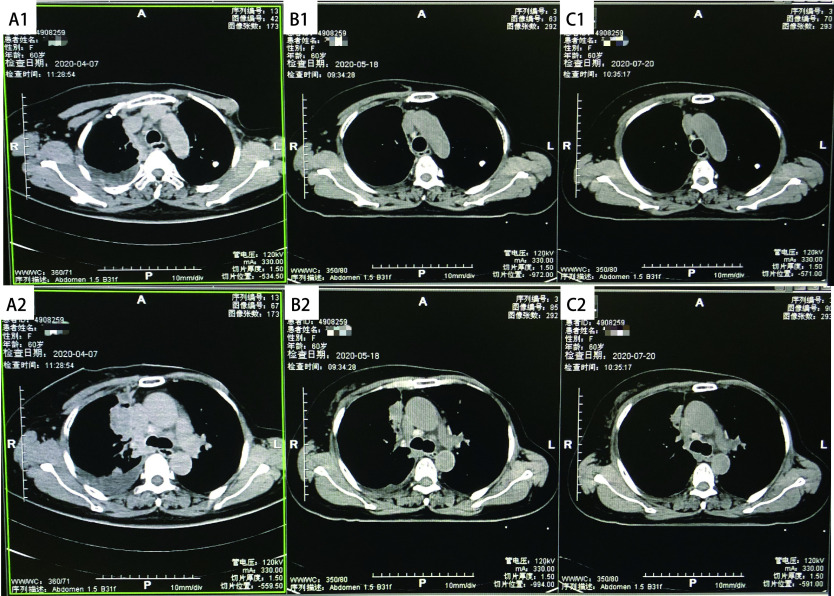
胸部CT扫描图像：A：2020年4月7日纵隔侧胸膜转移病灶（A1）及右肺上叶肿瘤病灶（A2）；B：2020年5月18日纵隔侧胸膜转移病灶较前明显缩小（B1）及右肺上叶肿瘤病灶较前明显缩小（B2）；C：2020年7月20日纵隔侧胸膜转移病灶基本消失（C1）及右肺上叶肿瘤病灶进一步缩小（C2）。 CT images of the chest. A: Mediastinal pleural metastases lesion (A1) and right upper lung tumor (A2)(Apr 7, 2020); B: Mediastinal pleural metastases lesion was significantly smaller than that in the former (B1) and right upper lung tumor was significantly smaller than that in the former (B2)(May 18, 2020); C: Mediastinal pleural metastases lesion was disappear (C1) and right upper lung tumor was shrinks further (C2)(Jul 20, 2020). CT: computed tomography.

## 2 讨论

肺癌是我国乃至全球发病率及死亡率均较高的恶性肿瘤，其中NSCLC占80%左右，在NSCLC中，约5%的NSCLC肿瘤中发现涉及2号染色体*ALK*基因位点的染色体重排^[1]^。ALK是一种跨膜受体酪氨酸激酶，属于胰岛素受体超家族，*ALK*融合基因是在研究间变大细胞淋巴瘤的基因分析中发现的，可在多种肿瘤中异常表达。NSCLC中最常见的*ALK*重排将*EML4*基因的5'端与*ALK*基因的3'端并列，产生了新的融合癌基因*EML4-ALK*^[2]^。这种融合癌基因产生的*EML4-ALK*融合蛋白可引起ALK的胞内激酶结构域发生二聚化，激活经典的ALK下游致癌信号，导致患者病情进展及较差的预后^[3]^。下游信号通路的表达促进了肿瘤的进展，其中下游信号通路包括PI3K/AKT/mTOR通路和RAS/MEK/ERK通路^[4, 5]^。ALK阳性NSCLC为独特的临床病理亚型。在新诊断的NSCLC患者中检测ALK基因重排是至关重要的，因为这种癌基因的存在会影响治疗决策。

在一项对303例被随机分为一线阿来替尼与克唑替尼（ALEX）的患者的全球研究^[6]^中，接受阿来替尼的患者进展或死亡风险降低了53%（HR=0.47, 95%CI: 0.34-0.65），中位无进展生存期（progression free survival, PFS）未达到，而接受克唑替尼的患者平均随访时间约为18个月。在全球ALEX研究的更新中，经过10个月的随访，阿来替尼组的平均PFS为35个月，而克唑替尼组为11个月（HR=0.43）^[7]^。总体生存（overall survival, OS）率结果尚不成熟。

在J-ALEX中，207例日本ALK阳性的NSCLC患者被随机分为阿来替尼组和克唑替尼组^[8]^。中期分析结果显示阿来替尼组的PFS得到改善（HR=0.34, *P* < 0.000, 1）；阿来替尼组的中位PFS未达到，而克唑替尼组中位PFS为10.2个月（HR=0.34, 95%CI: 0.17-0.70）。阿来替尼的耐受性也更好，最常见的不良反应是便秘（36%）。接受克唑替尼治疗的患者出现恶心（74%）、腹泻（73%）、视力障碍（55%）和丙氨酸转氨酶/天冬氨酸转氨酶升高（>30%）等毒性反应^[9]^。

在一项三期临床研究（ALESIA）^[10]^中，阿来替尼与克唑替尼在未经治疗的晚期ALK阳性NSCLC患者中进行了比较。共有187例患者按2:1的比例随机分配接受阿来替尼或克唑替尼。中位随访时间为15个月-16个月。与J-ALEX和ALEX相似，阿来替尼与进展或死亡风险的降低相关（HR=0.22, 95%CI: 0.13-0.38），阿来替尼组的中位PFS未达到，而克唑替尼组为11.1个月。根据PFS的独立评价，阿来替尼也优于克唑替尼（HR=0.37, 95%CI: 0.22-0.61）。在亚组分析中，与未发生脑转移的患者相比，阿来替尼对脑转移患者的获益显著高于克唑替尼（HR=0.11, 95%CI: 0.05-0.28）及无脑转移的患者（HR=0.34, 95%CI: 0.18-0.65）。阿来替尼组严重和3级-5级不良事件发生率低于克唑替尼。

基于上述临床试验，目前阿来替尼用于ALK阳性的局晚期或转移性NSCLC，包括一线及克唑替尼治疗进展后的二线治疗。

在肿瘤患者治疗中药物治疗不良反应是不容忽视的，药物治疗不良反应的及时发现、及早治疗能极大地提高患者生活质量，患者治疗依从性也会提高，从而达到更好的临床获益。患者在使用阿来替尼中出现胃肠道不良反应及贫血，目前公认的肿瘤相关贫血的主要机制：Spivak等^[11]^指出，EPO产生受抑为肿瘤患者癌性贫血的重要原因，认为肿瘤可以刺激炎性细胞因子产生，炎性细胞因子可以直接抑制红系祖细胞增殖，也可以抑制EPO的产生。其他机制：目前研究认为，肿瘤细胞和宿主免疫系统相互作用可致巨噬细胞活化，使γ-干扰素（interferon-γ, IFNγ）、白介素-1（interleukin-1, IL-1）、肿瘤坏死因子（tumor necrosis factor, TNF）等炎性细胞因子表达和分泌增加，这些因子增加可直接抑制EPO产生。目前治疗相关性贫血危害主要包括：①缺氧更易导致血管生成因子的产生，可能促肿瘤生长；②对治疗的耐受性降低，推迟治疗疗程，降低药物剂量；③因贫血引起缺氧及组织器官功能受损、头晕、疲劳乏力。该患者首先出现恶心、呕吐、进食量下降，后出现贫血，患者贫血也与其消化道不良反应相关，所以在治疗中我们给予止吐、保护胃黏膜对症治疗，在贫血方面给予EPO 10, 000 iu、皮下注射、每周3次，同时加强营养、铁剂、叶酸、维生素B12及抗凝治疗，治疗后患者恶心呕吐症状缓解，进食量增加，患者乏力症状缓解，血红蛋白逐渐升高，目前无明显不适主诉。

在不良反应管理中，主要包括预防、个体化、全程、整体性及多学科协作。①预防：主要是指我们在用药前要熟知所使用药物的常见不良反应，提前告知患者，必要时给予药物预防药物相关不良反应；②个体化：每位患者可能出现不良反应的种类、严重程度不同，所以我们的治疗需根据患者具体情况来制定；③全程：不良反应的管理是动态的、持续性的，根据患者病情随时调整；④整体性：患者可能存在多种不良反应，我们要从整体的观念来看待问题，分清主次，从而使治疗有条不紊；⑤多学科协作：在药物不良反应中可能出现其他科室相关疾病，如内分泌科（甲状腺功能异常）、消化科（药物性肝损害）、心内科（心律失常、心功能异常）等，常常需要多学协作，共同为患者制定合理、有效的治疗方案。
